# Proliferative Glomerulonephritis With Monoclonal Immunoglobulin G Deposits (PGNMID) With Chronic-Type Thrombotic Microangiopathy (TMA): A Case Report

**DOI:** 10.7759/cureus.91691

**Published:** 2025-09-05

**Authors:** Koji Yoshimoto, Kensuke Sasaki, Ryo Tamura, Akira Takahashi, Maria Yoshida, Yosuke Osaki, Naoki Ishiuchi, Aiko Okubo, Yujiro Maeoka, Shigeo Hara, Takao Masaki

**Affiliations:** 1 Department of Nephrology, Hiroshima University Hospital, Hiroshima, JPN; 2 Department of Pathology, Kobe City Medical Center General Hospital, Kobe, JPN

**Keywords:** biopsy, chronic thrombotic microangiopathy (tma), monoclonal gammopathy of renal significance (mgrs), proliferative glomerulonephritis with monoclonal igg deposits (pgnmid), steroid

## Abstract

Proliferative glomerulonephritis with monoclonal immunoglobulin G (IgG) deposits (PGNMID) is a form of monoclonal gammopathy of renal significance. A pathological evaluation of a kidney biopsy is essential for the diagnosis of PGNMID because it is characterized by glomerulonephritis with exclusively glomerular monotypic immunoglobulin deposits, which are typically IgG, and less commonly IgM or IgA. Membranoproliferative glomerulonephritis and endocapillary proliferative glomerulonephritis are the typical pathological features of PGNMID.

A 53-year-old Japanese woman presented with persistent proteinuria for more than 10 years. The patient had a medical history of unknown acute kidney injury 20 years previously and received a 1-year course of steroid therapy. She was later diagnosed with nephrosclerosis and received angiotensin II receptor blocker (ARB) therapy for over two years; however, her proteinuria gradually worsened, and renal function declined. A kidney biopsy revealed focal segmental endocapillary hypercellularity with mild mesangial proliferation and double contours of glomerular basement membranes. Immunofluorescence showed granular staining for IgG, C3, C1q, IgG3, and λ in the mesangial areas and along the glomerular capillary walls. Electron microscopy demonstrated electron-dense deposits without organized structures in the paramesangial and mesangial areas, together with subendothelial edema and new basement membrane formation. These findings did not conform to the typical membranoproliferative pattern, leading to a diagnosis of PGNMID with chronic thrombotic microangiopathy (TMA). In addition to ongoing ARB therapy, corticosteroid treatment reduced proteinuria and stabilized renal function. To our knowledge, this is a rare case of PGNMID that was accompanied by chronic TMA.

## Introduction

Proliferative glomerulonephritis with monoclonal immunoglobulin G (IgG) deposits (PGNMID) is a rare glomerular disease classified under monoclonal gammopathy of renal significance (MGRS), a group of renal disorders associated with underlying hematologic conditions [1]. MGRS encompasses kidney injuries caused by nephrotoxic monoclonal immunoglobulins produced by small B-cell or plasma cell clones, even in the absence of overt hematologic malignancy, and therefore has important implications for both renal and hematologic management. The incidence of PGNMID in renal biopsy series has been reported to range from 0.17% to 3.7% [2]. The detection rate of M-protein in patients with PGNMID is relatively low (approximately 30%) [2,3], making early diagnosis and disease recognition challenging. Clinically, the recognition of PGNMID is important because monoclonal gammopathies may indicate an underlying hematologic disorder, and distinguishing PGNMID from other immune complex-mediated glomerulonephritides has direct implications for prognosis and treatment. A definitive diagnosis is made by a renal biopsy, which typically shows glomerular deposition of monoclonal IgG restricted to a single IgG subclass and a single light chain subtype. Histologically, PGNMID most commonly presents with membranoproliferative glomerulonephritis (MPGN)-like features or endocapillary proliferative glomerulonephritis on light microscopy [1,2]. MPGN-like lesions are characterized by a glomerular hypercellularity, mesangial matrix expansion, and double contour formation of the glomerular basement membrane (GBM). Typically, double contour formation of the GBM in MPGN-like lesions is accompanied by significant mesangial proliferation; cases showing marked double contours with only mild mesangial changes are unusual.

Endothelial injury and complement activation have also been reported in PGNMID, suggesting possible overlap with the pathophysiology of thrombotic microangiopathy (TMA). While acute TMA is typically defined by thrombi and systemic manifestations, chronic kidney-limited endothelial injury may manifest primarily as persistent GBM remodeling with double contours, a broader concept that has been described as glomerular microangiopathy (GMA).

To our knowledge, reports of PGNMID accompanied by chronic TMA/GMA-like pathology are extremely rare. Here, we report a case of PGNMID presenting with focal segmental endocapillary hypercellularity and mild mesangial proliferation, along with marked double contours of GBM--a histological feature that cannot be fully explained by PGNMID alone and highlights the possible coexistence of chronic endothelial injury.

## Case presentation

History of present illness

A 53-year-old Japanese woman was admitted with persistent proteinuria, which had been present for more than 10 years, and progressive renal dysfunction. Approximately 20 years earlier, she had experienced an episode of acute kidney injury of unknown cause, for which she received a one-year course of steroid therapy. No kidney biopsy was performed at that time. Persistent proteinuria was not documented at that time. About 10 years later, proteinuria was first detected, and it gradually increased thereafter, despite treatment with an angiotensin II receptor blocker (ARB) initiated two years prior to admission.

Past medical history

The patient had previously been diagnosed with nephrosclerosis and treated with an ARB by her primary physician for the past two years. There was no family history of kidney disease.

Examination findings

On admission, her blood pressure was 126/73 mmHg, and physical examination revealed no abnormalities.

Investigations

The results of the laboratory tests were as follows: serum blood urea nitrogen, 7.96 mmol/L (reference: 2.9-8.2 mmol/L); serum creatinine (Cr), 106.96 µmol/L (reference: 44-106 µmol/L); C3, 0.87 g/L (reference: 0.73-1.39 g/L); C4, 0.25 g/L (reference: 0.11-0.41 g/L). Autoantibodies, including anti-nuclear, anti-neutrophil cytoplasmic, and anti-GBM, were negative (Table 1). Urinalysis revealed a urine protein-to-creatinine ratio of 348.71 mg/g and 1-4 red blood cells per high-power field. Computed tomography demonstrated atrophy of the left kidney with compensatory hypertrophy of the right kidney. The patient underwent a kidney biopsy of the right kidney to determine the underlying cause of proteinuria and renal dysfunction. The etiology of the left contracted kidney remains uncertain. There was no documented history of urinary tract infection, renal artery stenosis, or renal stones, and the length of the left kidney was not recorded. Histological evaluation for atherosclerotic lesions as a potential cause of left renal atrophy was not specifically performed, which is acknowledged as a limitation of this report.

**Table 1:** Laboratory results on admission RBC, red blood cells; HPF, high-power field; WBC, white blood cells; Hb, hemoglobin; Plt, platelets; Alb, albumin; UA, uric acid; BUN, blood urea nitrogen; Cr, creatinine; eGFR, estimated glomerular filtration rate; Na, sodium; K, potassium; Cl, chloride; Ca, calcium; P, phosphate; AST, aspartate transaminase; ALT, alanine transaminase; CRP, C-reactive protein; HbA1c, Hemoglobin A1c; NGSP, national glycohemoglobin standardization program; ANA, antinuclear antigen; MPO-ANCA, myeloperoxidase-neutrophilic cytoplasmic antibody; PR3-ANCA, proteinase 3 anti-neutrophilic cytoplasmic antibody; GBM, glomerular basement membrane; IgG, Immunoglobulin G; IgA, Immunoglobulin A; IgM, Immunoglobulin M; κ, kappa; λ, lambda; FLC, free light chain; HBs-Ag, hepatitis B virus antigen; HCV-Ab, hepatitis C virus antibody. * Out of reference range

Parameter	Unit	Value	Reference range
Urine			
pH	-	5	-
Urine protein/creatinine ratio	(mg/g)	348.71*	0–150
RBCs	(/HPF)	01-Apr	0–5
Urine protein electrophoresis	-	Negative	Negative
Blood			
WBCs	(×10⁹/L)	4.91	3.04–8.54
RBCs	(×10^12^/L)	3.44*	3.78–4.99
Hb	(g/L)	105*	108–149
Hematocrit	(/L)	0.33*	0.36–0.45
Plt	(×10^9^/L)	207	150–360
Biochemistry			
Total protein	(g/L)	69	67–83
Alb	(g/L)	43	40–50
UA	(μmol/L)	422.31*	136.8–416.36
BUN	(mmol/L)	7.96*	2.86–7.14
Cr	(μmol/L)	106.96*	35.36–61.88
eGFR	(mL/min/1.73m^2^)	37*	> 90
Na	(mmol/L)	142	138–146
K	(mmol/L)	4.6	3.6–4.9
Cl	(mmol/L)	110*	99–109
Ca	(mmol/L)	2.27	2.15–2.59
P	(mmol/L)	1.5	0.81–1.51
AST	(IU/L)	28	13–33
ALT	(IU/L)	21	8–42
CRP	(mg/L)	0.2	0–2.0
Plasma glucose	(mmol/L)	4.05	3.89–6.05
HbA1c (NGSP)	(%)	5.6	4.6–6.2
C3	(g/L)	0.87	0.86–1.6
C4	(g/L)	0.25	0.17–0.45
ANA	-	Negative	Negative
MPO-ANCA	-	Negative	Negative
PR3-ANCA	-	Negative	Negative
Anti-GBM antibody	-	Negative	Negative
IgG	(g/L)	9.25	8.7–17
IgA	(g/L)	1.94	1.1–4.1
IgM	(g/L)	1.31	0.46–2.6
Free κ light chain	(mg/L)	23.8*	2.42–18.92
Free λ light chain	(mg/L)	17.6	4.44–26.18
FLC ratio (κ/λ)	-	1.35	0.26–1.65
Serum protein electrophoresis	-	Negative	Negative
HBs-Ag	-	Negative	Negative
HCV-Ab	-	Negative	Negative

Kidney Biopsy

**Figure 1:**
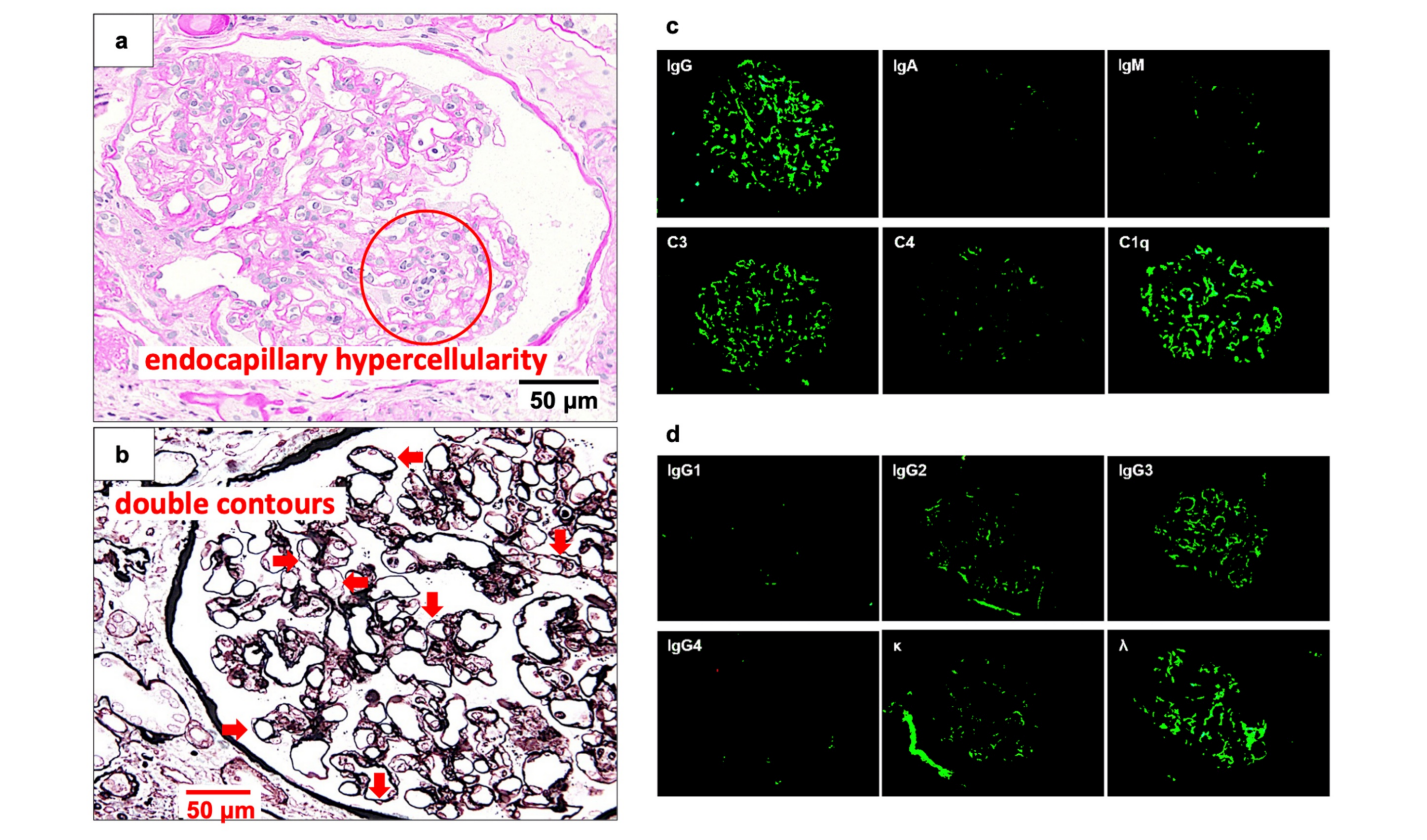
Light microscopy and immunofluorescence findings of the kidney biopsy (a) Periodic acid–Schiff staining showing segmental endocapillary hypercellularity (red circle). (b) Periodic acid-methenamine-silver staining demonstrating focal double contours of the glomerular basement membrane (GBM) without spike formation (arrows) and endocapillary hypercellularity. (c, d) Immunofluorescence images showing strong granular deposits of IgG (especially IgG3) and λ light chain, with moderate positivity for C3 and C1q, predominantly in the mesangial areas and along the capillary walls, consistent with monoclonal IgG3-λ deposition and supporting the diagnosis of PGNMID. Red circle, segmental endocapillary hypercellularity; Arrows, double contours

**Figure 2:**
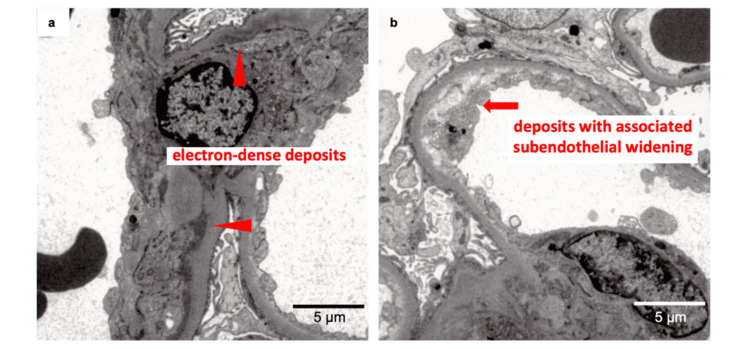
Electron microscopic images of the kidney biopsy (a, b) Electron microscopy showing electron-dense deposits in the paramesangial area to the mesangial area (arrowheads) and focal subendothelial deposits with associated subendothelial widening (arrows). Some areas exhibit podocyte foot process effacement. The deposits were moderate to marked in size and density, consistent with chronic immune complex deposition and chronic endothelial injury.

The renal specimen contained 12 glomeruli, 4 of which showed global sclerosis. Light microscopy showed focal and segmental endocapillary hypercellularity and mild mesangial proliferation (Figure 1). The glomerular basement membrane exhibited segmental double contours. No thrombi were observed. Congo red staining was negative. Immunofluorescence (IF) studies showed a granular staining pattern of IgG, C3, and C1q along the glomerular capillaries and within the mesangial areas. Further IF studies for κ/λ and the IgG subclass demonstrated monoclonal deposition of IgG3-λ in these regions. Electron microscopy revealed electron-dense deposits without an organized structure in the paramesangial and mesangial areas. No electron-dense deposits were detected in the subendothelial space. Subendothelial edema and newly formed basement membrane were observed (Figure 2). Podocyte foot processes exhibited diffuse effacement. No marked glomerular enlargement suggestive of compensatory hyperfiltration or secondary focal segmental glomerulosclerosis (FSGS) was observed, even though imaging demonstrated compensatory hypertrophy of the right kidney. Although GBM double contours alone are not pathognomonic for chronic type TMA, the coexistence of GBM double contours, mesangial expansion, subendothelial widening, endothelial swelling, and occasional fibrin deposition, together with chronic endothelial injury, in the absence of systemic TMA findings (no anemia, thrombocytopenia, or LDH elevation) and histological thrombi, is consistent with established pathological criteria for chronic type, kidney-limited TMA [4-6]. Based on these pathological findings and established criteria, the patient was diagnosed with PGNMID with monoclonal IgG3-λ deposits associated with chronic type, kidney-limited TMA.

Differential diagnosis

Based on the clinical history and laboratory findings, several alternative diagnoses were initially considered. Primary glomerulopathies, such as idiopathic membranoproliferative glomerulonephritis (immune-complex-mediated) and membranous nephropathy, were considered less likely because immunofluorescence revealed monoclonal IgG3-λ deposition rather than polyclonal immune deposits. Autoimmune diseases, such as lupus nephritis, were excluded by the absence of systemic symptoms and negative autoantibody tests. Secondary causes, such as hypertensive nephrosclerosis, were also considered, but the biopsy showed features of glomerular basement membrane double contours and monoclonal deposits inconsistent with this etiology. Taken together, these considerations supported the final diagnosis of PGNMID with coexisting chronic, kidney-limited TMA.

Clinical course

**Figure 3:**
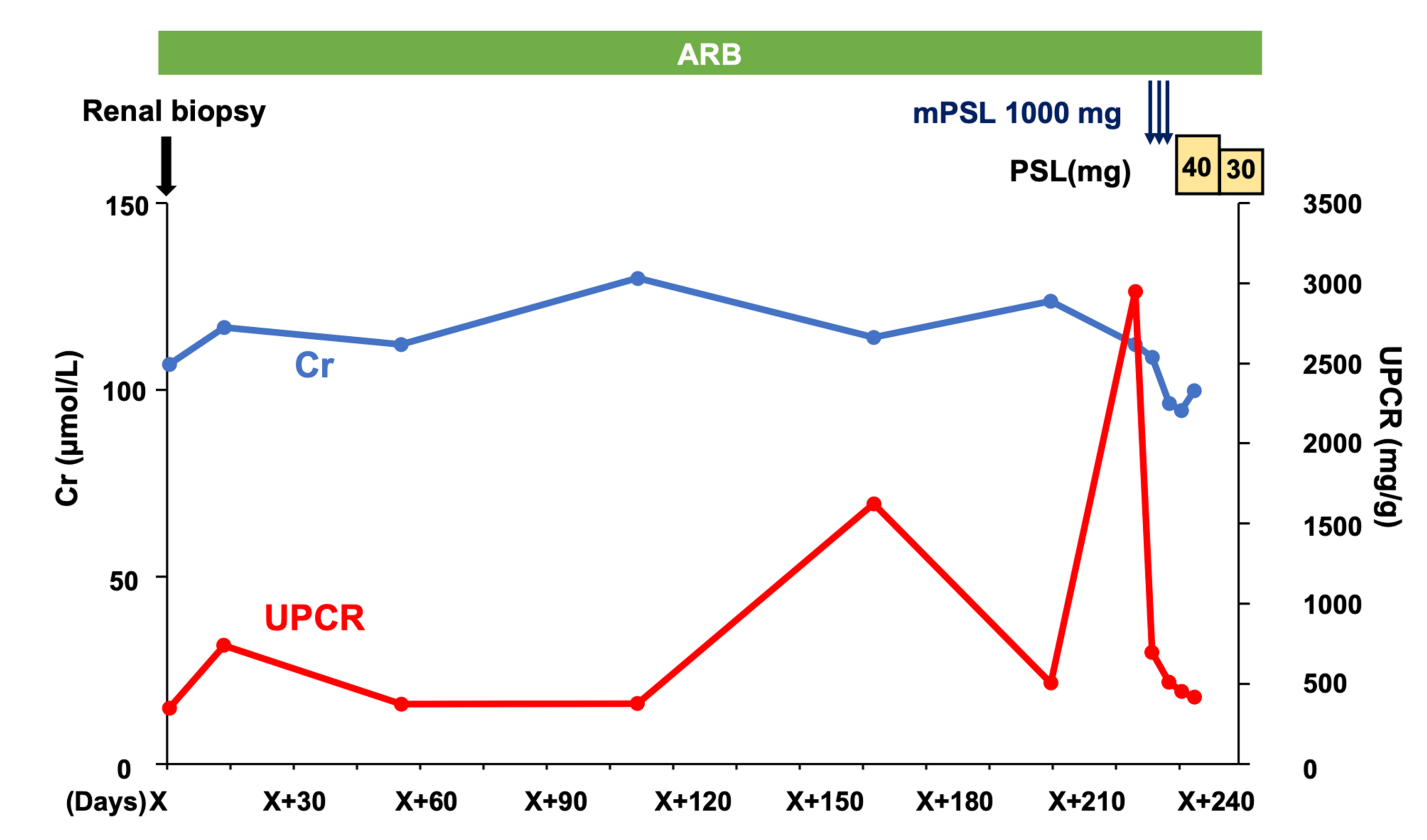
Clinical progression of the patient Graph showing changes in serum creatinine (Cr, blue line) and the urine protein-to-creatinine ratio (UPCR, red line) during follow-up. Despite continued treatment with an angiotensin II receptor blocker (ARB), UPCR increased progressively and rose sharply before intervention. Following a renal biopsy (X), the patient received methylprednisolone pulse therapy (mPSL, 1 g/day for 3 days) followed by oral prednisolone (PSL). This treatment led to a marked reduction in proteinuria and stabilization of renal function. X: date of the renal biopsy, ARB: angiotensin Ⅱ receptor blocker, mPSL: methylprednisolone, PSL: prednisolone, Cr: creatinine, UPCR: urine protein-to-creatinine ratio

Although treatment with an ARB was continued after the kidney biopsy, the urine protein-to-creatinine ratio gradually increased from 739.22 to 2947.54 mg/g. Consequently, the patient received intravenous methylprednisolone pulse therapy (1 g/day for 3 days), followed by oral prednisolone at a daily dose of 40 mg. Following the initiation of steroid treatment, the proteinuria gradually decreased to 415.89 mg/g (Figure 3).

## Discussion

We describe a rare case of PGNMID accompanied by chronic TMA, characterized by marked double contours of the GBM and mild mesangial proliferation. In our patient, serum and urine protein electrophoresis did not reveal an M-protein, and there were no hematologic abnormalities suggestive of a clonal disorder. Thus, a detailed discussion of MGRS-related TMA was considered less relevant in this case, and a bone marrow biopsy was not performed. We therefore focused on the pathological overlap between PGNMID and chronic TMA. Classical MPGN, defined by mesangial proliferation and double contours of GBM, is the most common pathological manifestation of PGNMID, followed by endocapillary proliferative glomerulonephritis [1,2]. In classical MPGN, subendothelial deposition of immunoglobulins induces double contour formation. In this case, however, although double contours were evident, mesangial proliferation was minimal, making classical MPGN less likely; instead, suggesting a diagnosis of chronic TMA. This diagnosis was based primarily on histopathological evidence of chronic endothelial injury, and although other causes, such as autoimmune disease, malignant hypertension, or drug-induced injury, were not supported by the patient’s history or laboratory data, additional investigations, including ADAMTS13 activity, complement profiling, and genetic testing, were not performed. Therefore, the interpretation should be regarded as somewhat speculative. In addition, double contours are often seen in proliferative glomerular diseases, such as MPGN, where it is usually accompanied by active intracapillary inflammation due to immune complex deposition and endothelial injury. However, in this case, although the glomerular inflammation was minimal, double contours were prominent and widely distributed. This dissociation between mild inflammatory activity and marked structural remodeling cannot be fully explained by PGNMID alone. These features are more consistent with a chronic endothelial injury pattern, as seen in chronic TMA, where GBM remodeling may persist even in the absence of overt thrombi or cellular proliferation. Differential diagnoses were also considered. Mesangial-dominant PGNMID was unlikely because immune deposits were not confined to the mesangial area but extended to the glomerular capillary walls. Atypical membranous nephropathy was excluded owing to the absence of subepithelial deposits and the predominance of mesangial and subendothelial lesions. Although chronic hypertensive nephrosclerosis could account for some vascular alterations, the presence of monoclonal IgG3-λ deposits and characteristic immune-complex-mediated pathology supported a final diagnosis of PGNMID with coexisting chronic TMA. Although no thrombi were observed, the prominent double contours with minimal mesangial proliferation suggested a chronic endothelial injury pattern. While we referred to this as “chronic TMA,” the broader concept of “GMA” may also be appropriate to describe the observed pathology. Clinical and laboratory evaluations did not reveal evidence of genetic TMA, complement-mediated disease, drug-induced TMA, or autoimmune conditions, and there were no systemic features of acute TMA such as microangiopathic hemolytic anemia or thrombocytopenia. Thus, the pathological findings were interpreted as PGNMID with coexisting chronic endothelial injury, most consistent with chronic kidney-limited TMA/GMA.

TMA is characterized by primary microvascular endothelial cell injury, leading to the occlusion of small vessels by platelets and/or fibrin thrombi, mainly in the kidney and brain [7,8]. One of the pathological features of chronic TMA on light microscopy is double contouring, resulting from subendothelial expansion with mesangial interposition [9,10]. IF and electron microscopy are essential to distinguish between these conditions. In our case, based on light microscopic findings and a medical history of unspecified acute kidney injury and hypertension, we initially suspected that the persistent proteinuria and decline in renal function were due to chronic TMA and nephrosclerosis. However, IF revealed a granular staining pattern of IgG3-λ in the capillary loops and mesangial regions. Moreover, electron-dense deposits and macrophage infiltration were identified in the paramesangial and mesangial areas. Based on these findings, we diagnosed this case as early-stage PGNMID with monoclonal IgG3-λ deposits associated with chronic TMA. To our knowledge, there have been no previous reports of PGNMID with chronic TMA-like findings on light microscopy.

Chronic TMA is characterized by persistent proteinuria and progressive deterioration of kidney function; however, its underlying etiology and pathogenesis remain poorly understood [9]. Pathologically, chronic TMA is distinguished by double contouring of glomerular capillary walls caused by the formation of new basement membrane beneath damaged endothelium. In some cases, the arterial walls exhibit an “onion-skin” appearance due to concentric intimal and medial hyperplasia [6]. TMA commonly results from primary microvascular endothelial injury, which leads to the occlusion of small vessels by platelet and/or fibrin thrombi. These conditions are often accompanied by systemic features, such as microangiopathic hemolytic anemia, thrombocytopenia, and thrombotic acute kidney injury (AKI), and are generally referred to as "acute TMA." However, systemic manifestations are not required for the diagnosis of TMA. In fact, kidney-predominant TMA is commonly encountered in clinical practice [6]. Therefore, when GBM double contours are observed without nephrotic-range proteinuria, the possibility of chronic TMA should be considered. In PGNMID cases like the present one, where marked GBM double contours are present despite only mild mesangial proliferation and segmental endocapillary hypercellularity--a finding not fully accounted for by MPGN pathophysiology--the coexistence of chronic TMA should be suspected. Finally, persistent proteinuria lasting for more than 10 years before the diagnosis of PGNMID possibly reflects the preceding chronic TMA.

We report a rare case of PGNMID that presented with chronic TMA-like pathology, characterized by marked double contours of the GBM and mild mesangial proliferation on light microscopy. This case exhibited persistent proteinuria and residual GBM duplication, despite the mild glomerular inflammation on histological examination. If the previous PGNMID had not completely resolved and was still ongoing, these GBM changes would be expected to show improvement rather than remain as prominent as observed in this case. Therefore, we considered the possibility that another disease might be contributing to or coexisting with PGNMID in this patient. We diagnosed this case as early-stage PGNMID with monoclonal IgG3-λ deposits accompanied by chronic TMA, based on findings from light microscopy, immunofluorescence staining, and electron microscopy. Finally, steroid therapy successfully reduced the patient’s proteinuria and stabilized renal function.

PGNMID, which is a type of kidney disease showing the classical MPGN pattern, is defined as a novel form of glomerular injury related to monoclonal IgG deposition [2]. The reported incidence of PGNMID in renal biopsies ranges from 0.17% to 3.7% [2,11]. The clinical features of PGNMID were reported in a series of 37 patients [2]. Most of these patients were White female adults with a mean age of 54.5 years. All patients presented with proteinuria, 49% had nephrotic syndrome, and 68% had renal dysfunction. PGNMID has several histological features under light microscopy that have been discussed in relation to renal prognosis. The most frequent histological pattern of PGNMID is the membranoproliferative pattern (MPGN, 56.8%), followed by the endocapillary proliferative pattern (35.1%) and the crescentic pattern (32.4%). The membranous pattern (5.4%) and mesangial proliferative pattern (2.7%) are relatively rare [2]. On IF staining, the glomerular deposits are monoclonal and stain for a single light-chain isotype and a single γ heavy-chain subclass. Electron microscopy shows granular electron-dense deposits, mimicking ordinary immune-complex glomerulonephritis, in the mesangial and subendothelial areas. Recently, temporal changes in the pathological findings of PGNMID during disease progression have been reported [12,13]. Under light microscopy, minimal glomerular changes were observed in the early stage. As PGNMID progressed, mesangial proliferation and macrophage infiltration appeared, resulting in the MPGN pattern. On electron microscopy, electron-dense deposits were first detected in the mesangial region and subsequently appeared in the subendothelial region. In our case, light microscopy showed focal and segmental endocapillary hypercellularity and mild mesangial proliferation. Additionally, the location of electron-dense deposits was limited to the paramesangial and mesangial areas, and only mesangial interposition was detected in the subendothelial area. Although IF showed a fringe pattern staining, which is typical for MPGN, we diagnosed the patient as having the early stage of PGNMID based on the light microscopic and electron microscopic findings. The patient had previously received steroid therapy for acute kidney injury of an unknown cause. This finding suggests the presence of pre-existing chronic TMA, which, in addition to the development of PGNMID, may have contributed to the increased proteinuria.

There is no established treatment for PGNMID, but the effectiveness of steroid therapy has been reported in some cases [14-16]. Among 32 patients with a follow-up period of 30.3 months, 56% received steroids or immunosuppressant drugs, while 30% were treated with renin-angiotensin system inhibitors alone [2]. Regarding outcomes, 37.5% of the patients achieved complete or partial remission, another 37.5% experienced sustained deterioration of renal function, and 21.9% progressed to end-stage renal failure. In our case, the patient initially continued to receive an ARB, but her urinary protein levels increased. We therefore initiated intravenous methylprednisolone pulse therapy (1 g/day for 3 days), followed by oral prednisolone. This decision was based on the rapid progression of proteinuria despite ARB therapy and the coexistence of active glomerular lesions with chronic pathological changes, for which we considered that a strong initial immunosuppressive effect was needed. While proteinuria subsequently decreased and renal function stabilized in temporal association with steroid therapy, we acknowledge that the natural history of PGNMID, ongoing ARB treatment, or other unmeasured factors may also have contributed. Thus, the therapeutic effect of steroids in this case should be interpreted with caution. Although oral steroid therapy alone might have been an option, we considered that the combination of short-term pulse therapy and oral tapering provided the optimal balance between efficacy and safety in this clinical context. Furthermore, recent studies have shown promising results for daratumumab in the treatment of PGNMID. In a phase 2 study, daratumumab significantly improved proteinuria while stabilizing kidney function in patients with PGNMID [17].

Our report has several limitations. One possible cause of the chronic TMA is an episode of acute kidney injury of unknown etiology that occurred approximately 20 years ago; however, this remains purely speculative. Furthermore, this case lacks definitive clinical laboratory findings to confirm the coexistence of chronic TMA, and the renal biopsy did not reveal thrombi typically seen in acute TMA. Nevertheless, we report that the TMA coexisting with PGNMID in this case is not typical acute TMA but rather chronic TMA localized to the kidney. Although there have been reports of PGNMID undergoing spontaneous remission and subsequently relapsing after infectious exposures, such as acute upper respiratory tract infections or vaccinations [18], neither of these factors were present in our case.

## Conclusions

We report a case of PGNMID with pathological features of chronic TMA. The diagnosis of PGNMID was established based on a combination of light microscopy, IF, and electron microscopy. Further studies are required to clarify the underlying pathogenesis of PGNMID and to develop appropriate treatment strategies.
